# 

**Published:** 1893-03

**Authors:** 


### CONTENTS:

ORIGINAL ARTICLES: PAGE

Agricultural Shows, Judges and the Veterinarian. By R. S. Huidekoper, M.D., Veterinarian ... 129
Differential Diagnosis between Osteo Porosis and Rheumatism in Horses. By James D. Hopkins, V. S... 141
Bronchitis in Dogs. By R. L. Tucker, D.V.S... 144
Acute Toxic Anæmia. By Dr. Jacob Helmer... 150
Fungus Hæmatodes in Cattle and Horses, with Notes on Cases in Practice. By James A. Waugh, V.S... 156
Open Joints. By Dr. J. C. Michener... 160
Actinomycosis Bovis, or "Lump Jaw." By R. R. Dinwiddie, V. S... 163
Correction... 181

EDITORIAL:

Pennsylvania State Veterinary Medical Association... 182

SOCIETY PROCEEDINGS:

Keystone Veterinary Medical Association... 183
Indiana Association of Veterinary Graduates... 184
The Montreal Veterinary Medical Association... 186
The Montreal Veterinary Medical Association ... 188
The Montreal Veterinary Medical Association... 190
New Jersey State Veterinary Society... 192

